# Mercury and Other Biomedical Waste Management Practices among Dental Practitioners in India

**DOI:** 10.1155/2014/272750

**Published:** 2014-08-04

**Authors:** Raghuwar D. Singh, Sunit K. Jurel, Shuchi Tripathi, Kaushal K. Agrawal, Reema Kumari

**Affiliations:** ^1^Department of Prosthodontics, Faculty of Dental Sciences, King George's Medical University, Lucknow, Uttar Pradesh, India; ^2^Department of Community Medicine & Public Health, King George's Medical University, Lucknow, Uttar Pradesh, India

## Abstract

*Objectives*. The objective of the study was to assess the awareness and performance towards dental waste including mercury management policy and practices among the dental practitioners in North India. *Materials and Methods*. An epidemiologic survey was conducted among 200 private dental practitioners. The survey form was composed of 29 self-administered questions frame based on knowledge, attitude, and those regarding the practices of dentists in relation to dental health-care waste management. The resulting data were coded and a statistical analysis was done. *Results and Discussion*. About 63.7% of the dentists were not aware of the different categories of biomedical waste generated in their clinics. Only 31.9% of the dentists correctly said that outdated and contaminated drugs come under cytotoxic waste. 46.2% said they break the needle and dispose of it and only 21.9% use needle burner to destroy it. 45.0% of the dentists dispose of the developer and fixer solutions by letting them into the sewer, 49.4% of them dilute the solutions and let them into sewer and only 5.6% return them to the supplier. About 40.6% of the dentists dispose of excess silver amalgam by throwing it into common bin. *Conclusion*. It was concluded that not all dentists were aware of the risks they were exposed to and only half of them observe infection control practices.

## 1. Introduction

Management of waste generated in any health-care facility is a critical issue as it poses a direct threat to human health as well as to the environment. Dental setup is a multidisciplinary system which consumes lot of items for delivery of dental care [1]. With the advances in technology, many improved materials have emerged in the recent past. Many chemicals like acrylics, impression materials, and mercury used for restorative purposes may have a possible environmental and human health impact if not handled properly.

With the increase in demand for dental care, there has been a rapid growth of dental clinics in the recent years and this led to the increase in the amount of biomedical waste generated by them [2]. This has increased the incidence of nosocomial infections and environmental pollution leading to possibility of many diseases.

The biomedical waste generated in the dental scenario includes sharps, used disposable items, infectious waste (blood-soaked cotton, gauze, etc.), hazardous waste (mercury, lead), and chemical waste (such as spent film developers, fixers, and disinfectants). A major concern in our field is management and disposal of mercury. Mercury (Hg) as amalgam has been used as a direct restorative material for more than 15 decades [3]. Dentists and the dental personnel have been directly and indirectly exposed to Hg emissions from incinerators and Hg in waste water from the different sources which could be either from households or dental clinics [4]. The release of amalgam particles into dental office wastewater or in solid waste is an important concern as these particles could then be released into the environment [5]. These releases take part in the environmental pollution through direct wastewater, incineration, land-filling, and sewage sludge incineration, although the release from dentistry is less than 1% of the total mercury discharged annually into the environment as a result of mankind activities. Out of the 10000 tons of mercury released by industry, approximately 300 tons were contributed by dentistry all over the world in 1973 [6]. Accordingly, dental clinics are playing a major role in mercury discharge [7, 8]. If the manipulation of amalgam and its waste products are not strictly regulated, it could be responsible for environmental pollution as well as occupational exposure [9, 10].

To protect the environment and community from these hazards, the Ministry of Environment and Forest, Government of India, issued a notification on biomedical waste (management and handling) rules 1998 under Environment (protection) Act [11]. So it is the duty of every occupier of a hospital or clinic generating biomedical waste to take necessary steps to ensure that such waste is handled without any adverse effect to the human health or environment. Dental health-care setups are found to generate both infectious and hazardous waste, so it is the time for us to get oriented, sensitized, and trained to manage health-care wastes scientifically [12]. The present study is an effort to know the awareness and practices of dental care waste including mercury management among dental practitioners in two north Indian cities, namely, Lucknow and Kanpur. The objective of the study was to assess the awareness and performance towards dental waste including mercury management policy and practices among the dental practitioners.

## 2. Materials and Methods

An epidemiologic survey was conducted to assess the awareness and practices of biomedical and mercury waste disposal among dental practitioners in two north Indian cities. The study was cross-sectional and the source of data was primary. The survey form was composed of 29 self-administered questions framed based on knowledge, attitude, and those regarding the practice of dentists in relation to dental health-care waste management. The questionnaire also covered the procedure to control the waste amalgam and dispensing form used by the hospital. The questionnaire was designed in an appropriate way such that the objectives of the study were met. The questionnaire was distributed among 200 private dental practitioners at various dental clinics in two major north Indian cities.

The first section of the questionnaire consisted of the questions related to respondent's age, sex, qualification, and clinic location. Respondent's name was not recorded in order to ensure anonymity. The second section consisted of questions related to the awareness and practice of dental-care waste management. The questionnaire was pilot rested on a small group of dentists who were requested to complete it and to indicate any questions that they found unclear.

The dentists were approached personally or through e-mail. The questionnaire was distributed to them by the investigator and all the questions were explained to avoid any ambiguity. They were assured of the confidentiality of their responses and were requested to give appropriate answers.

The resulting data was coded and statistical analysis was done using SPSS (statistical package for social sciences) software version 17.0. Mean is calculated for demographic variables and percentages were calculated for the responses given by the dentist.

## 3. Results

A survey was conducted among 200 private dental practitioners with a self-administered questionnaire, out of which 160 responded (80%). The age of the participants ranged from 28 to 59 with the mean age of 32.5. Out of 160 participants, 139 (86.9%) were males and 21 (13.1%) were females. Ninety-seven participants (60.6%) completed postgraduation studies and 63 (39.4%) were undergraduates. Of the participants, 85 (53.1%) had been practicing for less than 5 years, 64 (40%) from 6 to 10 years, and 11 (6.8%) for more than 10 years.

About 63.7% of the dentists were not aware of the different categories of biomedical waste generated in their clinic. When asked about the category of an extracted tooth, 61.9% correctly said that it comes under the category of infectious waste. About 38.7% said that they do not know the category of the used needles or syringes, and only 23.7% correctly said that it comes under category 4 (waste sharps). Only 31.9% of the dentist correctly said that outdated and contaminated drugs come under cytotoxic waste.

**Figure 1:**
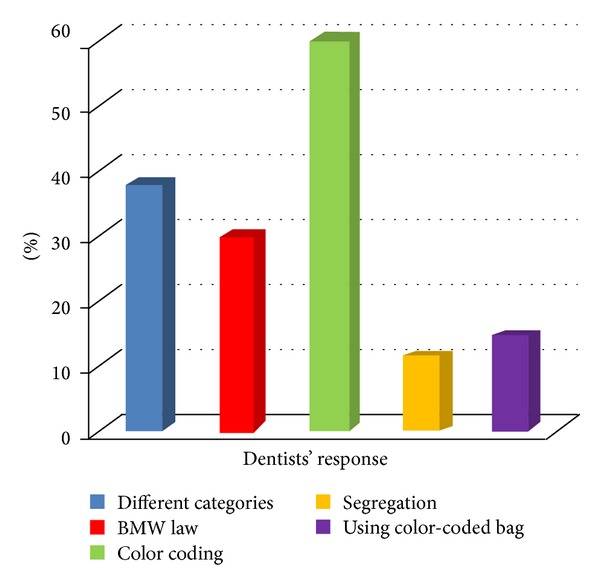
Awareness about biomedical waste management.

With regard to the question about the category of used cotton and impression materials, 39.4% rightly said that it falls under soiled waste.  Figure 1 shows awareness about biomedical waste management among dentists. Only 29.4% correctly said that human anatomical waste should be disposed of in yellow color container and 51.2% said they do not know. When being asked about the color coding for disposing sharp wastes, about 36.9% said they do not know and only 20.6% said it should be disposed of in blue/white translucent container.

**Figure 2:**
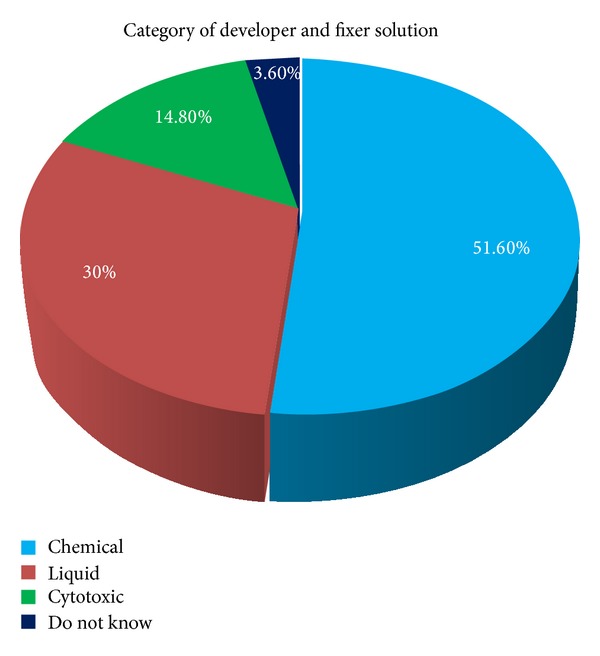
Dentists' response regarding category of developer and fixer solution.

Figure 2 describes the response of the dentists regarding the category of developer and fixer solution. Regarding the question about color coding for outdated and contaminated medicines, about 43.1% said they do not know and only 21.9% correctly said that it should be disposed of in a black container.

For the question regarding the disposal of sharp wastes like needle, 46.2% said they break the needle and disposed of it and only 21.9% used needle burner to destroy it, which was the ideal method. 45.0% of the dentists get rid of the developer and fixer solutions by letting them into sewer, 49.4% of dentists dilute them and pour them into sewer, and only 5.6% return them to the supplier. Nearly 68.1% of the dental practitioners get rid of the lead foil in the common bin, 29.4% stored separately and get rid of it, and only 2.5% return it to the certified buyers.

**Figure 3:**
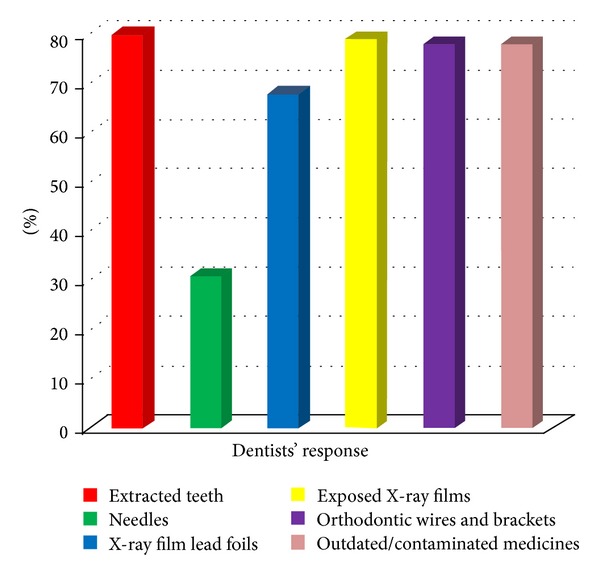
Disposal of dental wastes using common bin.

**Figure 4:**
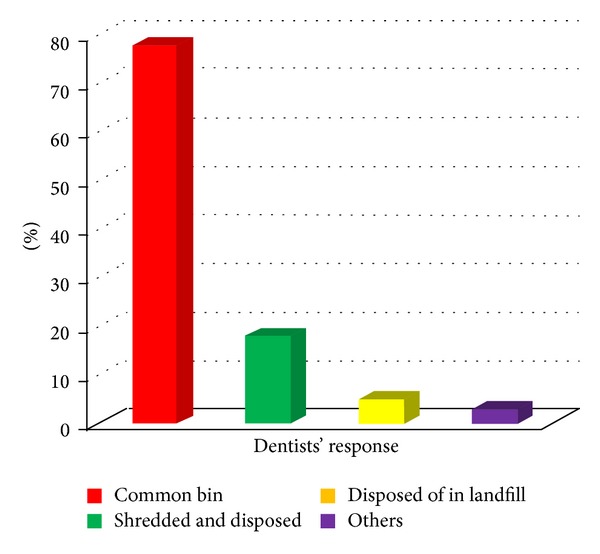
Methods of disposing plaster casts.

**Figure 5:**
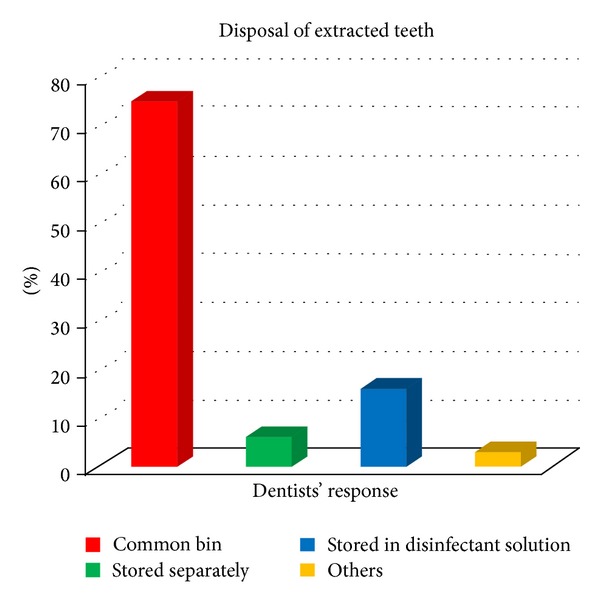
Methods of disposing extracted teeth.

**Figure 6:**
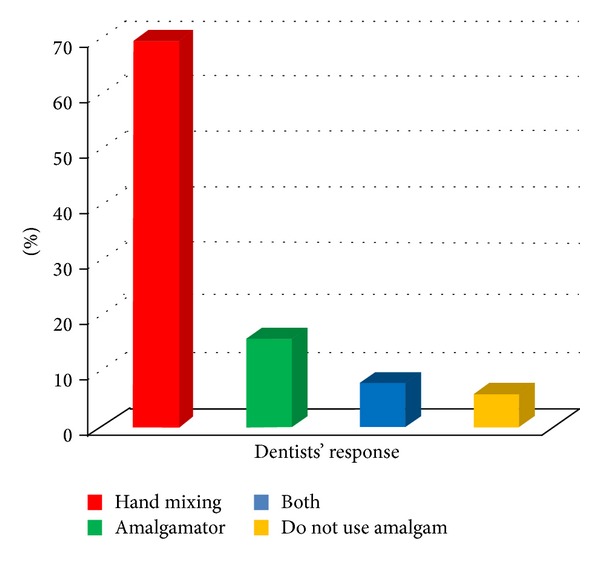
Dispensing form of amalgam used by dentists.

It was found that most of the dentists used common bin for the disposal of the different dental wastes as shown in Figure 3. Figures 4 and 5 give the responses of dentists regarding disposal of plaster casts and extracted teeth, respectively.  Figure 6 shows the dispensing form of amalgam used by the dentists. About 40.6% of the dentists dispose of excess silver amalgam into common bin (Table 1). The numbers of amalgam fillings performed and removed by the dentists per month were 62% and 38%, respectively.

## 4. Discussion

The main issue of concern in a dental practice is management of mercury. Silver amalgam has been used as dental restorative material for more than 150 years. Even today, with the advent of new synthetic nonmetallic materials and novel, time-saving procedures, silver amalgam is the most widely used and cost-effective dental material in restorative dentistry.

Mercury containing waste can be in form of elemental mercury or scrap amalgam (contact or noncontact amalgam scrap) [11]. Contact amalgam is amalgam that has been in contact with the patient, for example, extracted teeth with amalgam restorations; carving scrap collected at chair-side; and amalgam captured by chair-side traps, filters, or screens. Noncontact amalgam is amalgam that has not been in contact with the patient, for example, excess unused set amalgam and amalgam capsules. Both the contact amalgam and noncontact amalgam should be stored separately in different containers. The containers should be labelled with a “biohazard” symbol. As recommended by American Dental Association (ADA) guidelines should be followed for proper disposal of amalgam waste [11].

Placement and removal of dental amalgam restorations generate amalgam waste particles that are suctioned into vacuum line and discharged into sewer system. Chair-side traps and vacuum pump filters generally remove 40–80% of the amalgam particles from the wastewater stream; however, some amalgam particles still enter into the sewer system. Amalgam separators are devices designed to remove amalgam waste particles completely in dental office discharge [5, 13]. These separators remove the particles using different techniques such as sedimentation, filtration, centrifugation, or ion exchange. According to ADA, mercury and silver that are present in amalgam wastes should be recovered through a distillation process and sent for recycling. Amalgam that is rinsed down the drain may be released directly to a waterway, or it may be released into the air or into the soil. When the amalgam scrap is discarded along with the regular trash or along with the waste to be incinerated (yellow bag), mercury releases into the air or leaches into the groundwater.

A large number of dentists preferred to use the handmixing dispensation because of cost saving, which may increase the chances of handling error like improper mixing ratio that contains more mercury. Environ's study showed that 29.7 tons of mercury discharges into the wastewater system and only 0.4 ton of this mercury actually reaches surface waters in the United States annually [14], and another study conducted in New Delhi, India, revealed that 51 kg of mercury released amalgam waste each year from hospital and dental clinics [15].

**Figure 7:**
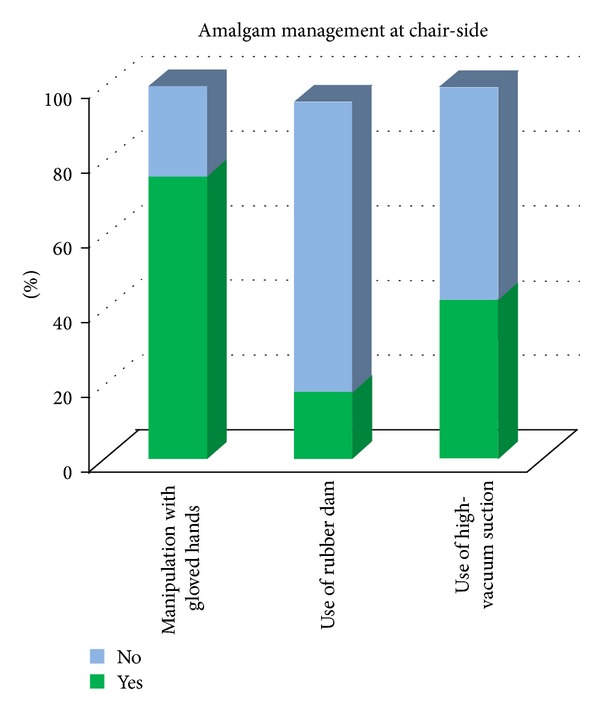
Dentists' performance during amalgam manipulation.

Forty-two percent of the dentists recommend replacement of an old amalgam restoration with the composite. In our study, 18% of the respondents use amalgamator to mix amalgam and 68% mix manually, whereas 5% are not using amalgam at all (Figure 6). 24% of the respondents were manipulating amalgam with ungloved hands, 78% do not use rubber dam while placing or removing amalgam restorations, and 57% do not use high-vacuum suction while handling amalgam in mouth (Figure 7). 47% of dentists were using cotton to hold excess Hg spilled on the floor, and 39% use stiff paper to pick it up.

In the present study, 30.6% of the dentists were aware of the biomedical waste management and handling law in India, while in a study conducted by Sudhakar et al. [16] in Bangalore and Kishore et al. [15] in New Delhi, only 57% and 36%, respectively, were aware of the biomedical waste management and handling law in India. This shows awareness of biomedical waste management law varies between cities.

When asked about the colour coding for different categories of biomedical waste 40.6% said they are not aware, while a study conducted in Davangere [2] showed that 27.2% are not aware. Majority of the dentists were actually not aware of the different categories of biomedical waste, although 36.3% said they were aware. The same hold true for the colour coding of biomedical waste.

In the present study, about 86.2% of the dentists do not segregate the wastes generated in their clinic which is similar to the study conducted by Sudhir [2] and Al-Khatib et al. [17], but in contrast to the study conducted by Sudhakar and Chandrashekar [16] in which only 35.7% do not practice segregation, 40.6% of the dentists get rid of the excess silver amalgam into common bin which is similar to the study conducted by Sudhakar and Chandrashekar [16] and Al-Khatib et al. [17], but in the study conducted by Sudhir [2], only 11.3% of the dentists dispose of it into common bin. Among 6.8% dentists who marked others, 5.6% were not using amalgam in their clinical practice and only 1.2% stored it in a fixer solution which is the recommended method by ADA. Empty amalgam capsules are to be disposed of in the garbage. Since amalgam decomposes on heating, it should not be incinerated [18].

31.9% of the dentists dispose of the used injection needles by throwing them into common bin and 46.2% break the needle and dispose of it, but in a study conducted by E. T. Treasure and P. Treasure [19] in New Zealand, only 24.4% dispose of it by throwing into common bin. In our study, the same 21.9% dentists use a needle destroyer to dispose of it which is the ideal method. It is of note that in both New Zealand and India there is legislation to ensure the proper disposal of clinical waste.

It was noted that 45.0% dispose of the developer and fixer solutions by letting them into sewer which is similar to a study conducted by Darwish and Al-Khatib [20] in Palestine. Developer solution does not contain silver, so it can be diluted and put into sewer; on the other hand fixer solution contains silver and if put into sewer it will increase the metal load in the sewer which is not allowed as per environmental protection rules. Spent fixer solution contains approximately 4000 mg of silver recovery units as reclaim silver. We have to store it separately and handle it over to certified buyers who will extract silver from it.

About 68.1% dispose of the X-ray film foils into common bin which is not permitted because lead is a heavy metal that affects neurological development and functions. It should not be incinerated or treated as general waste. It potentially leaches from landfills and can contaminate soil and ground water. Some of the factories may use lead as a raw material for manufacture of batteries, but the quantity required is high [18].

Only 15.6% stored exposed X-ray films separately which is in contrast to the study conducted by Sudhir et al. [2] in which half (52.9%) of the dentists store it separately. Exposed X-ray films are harmless and can be considered as general wastes. 77.5% dispose of orthodontic wires and brackets into common bin. According to OSHA (Occupational Safety and Health Administration) regulations, orthodontic wires are considered as sharp wastes because the ends of orthodontic wires can penetrate the skin and their contamination with blood can reasonably be anticipated. So they should be disposed as sharp wastes. Orthodontic brackets should be categorized as recyclable waste [21].

In the present study, 77.5% of the dentists dispose of outdated and contaminated medicines into common. They are considered as cytotoxic wastes and should be disposed of in a secured landfill [12]. 81.2% dispose of extracted teeth in common bin. OSHA considers extracted teeth to be potentially infectious material that should be disposed of in medical waste container. Extracted teeth which were sent to the dental laboratory for shade or size comparison, should be cleaned and surface-disinfected with a hospital disinfectant solution. Extracted teeth used for preclinical exercise should be autoclaved before using because liquid chemical germicides do not reliably disinfect both external surface and interior pulp tissue [21]. 15.6% of the dentist used colour-coded bags for the disposal of waste in their clinics and only 8.1% disposed of their dental wastes by returning to certified collectors, whereas in the study conducted by Sudhaker et al. [16] about 33.4% handle it over to certified agencies.

The validity and reliability of questionnaire-based surveys can be influenced by design, question content, analysis, and response rates. The advantages of using a questionnaire as a data collecting method was the quickly and inexpensive response from the respondents [16].

## 5. Conclusion

Within the limitations of the study, it can be concluded that not all dentists were aware of the risks they were exposed to and only half of them observed infection control practices. In addition to this, the majority of them were not aware of proper hospital waste management. A large proportion of the dentists were not practising proper methods of health-care waste disposal. Hence there is a need to educate the dental practitioners regarding proper practice measures.

## Figures and Tables

**Table 1:** Storage of excess silver amalgam.

Serial number	Storage of excess silver amalgam	Dentist response
1	Common bin	40.6%
2	Air-tight container with water	25.6%
3	Air-tight container	27%
4	Others	6.8%
